# Accessing the Climate Change Impacts in China through a Literature Mapping

**DOI:** 10.3390/ijerph192013411

**Published:** 2022-10-17

**Authors:** Keke Li, Bofeng Cai, Zhen Wang

**Affiliations:** 1 College of Resource and Environment, Huazhong Agricultural University, Wuhan 430070, China; 2 Center for Climate Change and Environmental Policy, Chinese Academy of Environmental Planning, Beijing 100012, China; 3 Interdisciplinary Research Center for Territorial Spatial Governance and Green Development, Huazhong Agricultural University, Wuhan 430070, China

**Keywords:** text-based classification, climate change adaption, temperature change, precipitation change

## Abstract

In the 21st century, carbon dioxide emissions have led to adverse climate changes; meanwhile, the impact of climate change has imposed challenges worldwide, particularly in developing countries, and China is one of the most affected countries. Assessing the impact of climate change requires handling a large amount of data in the literature comprehensively. In this study, a text-based classification method and literature mapping were used to process the massive literature and map it according to its location. A total of 39,339 Chinese academic studies and 36,584 Chinese master’s and doctoral theses, from 2000 to 2022, with evidence of the impact of climate change were extracted from the China National Knowledge Infrastructure database. Our results show that the literature on climate change impacts has exploded during the last decades. This indicates that increasing attention to the intensified impact of climate change in China has been paid. More importantly, by mapping the geolocation of the literature into spatial grid data, our results show that over 36.09% of the land area shows clear evidence of climate change. Those areas contribute to 89.29% of the gross domestic product (GDP) and comprise 85.06% of the population in China. Furthermore, the studies we collected on the climate change impacts showed a huge spatial heterogeneity. The hotspot areas of research were generally located in developed regions, such as the BTH urban agglomeration and Yangtze River Economic Zone, major agricultural production areas such as Shandong and Henan, and ecologically fragile regions including Yunnan, Xinjiang, and Inner Mongolia. Considering the imbalance spatially of the evidence of climate change can help in a better understanding of the challenges in China imposed by climate change. Appraising the evidence of climate change is of great significance for adapting to climate change, which is closely related to the natural ecosystem services and human health. This study will provide policy implications for coping with climatic events and guide future research.

## 1. Introduction

### 1.1. Background

Overwhelming evidence indicates that the atmospheric concentrations of key greenhouse gases increased and exacerbated the impacts of climate change over the 20th century. Future carbon dioxide emissions in the 21st century will continue to lead to adverse climate changes [1]. Meanwhile, natural and human systems are increasingly affected by climate change [2,3,4]. For example, climate change will increase the risk of species extinction [5], destroy marine ecosystems [6], exert negative effects on water resources and crop production [7,8], and threaten human health [9]. Climate change is a major threat in the 21st century [10]. Nevertheless, it has been confirmed that developing countries often suffer more severe consequences from climate change [2]. China, as the largest developing country, has experienced a notably changing climate [11]. Over the past few decades, the annual mean temperature in China has risen above the global average, and extreme climatic events, such as drought and flood, are occurring more frequently [12]. It has been projected that climate change will continue to intensify in the future [13]. The Ministry of Ecology and the Environment of the People’s Republic of China recently launched the policy paper “National Climate Change Adaptation Strategy 2035”, which urges building a regional pattern of adaptation to climate change and effectively addresses the adverse impacts of climate change [14]. Thus, how to adapt to climate change has become a priority for China [15].

### 1.2. Literature Review

Over the past few decades, the scientific community has contributed massively to thediscussions and predictions regarding climate change [15,16]. Exponential growth in published climate change research has been witnessed, and this trend is likely to continue [17]. The number of journals dedicated primarily or exclusively to climate change research has soared [17]. Furthermore, the assessment of the Intergovernmental Panel on Climate Change showed an increase of greater than two orders of magnitude in the number of relevant studies concerning the impact of climate change [2]. Consequently, fully utilizing the growth in the literature and the available information on the impact of climate change will help mitigate the future effects [18]. However, the expansion of the scientific literature on climate change has increased the amount of manual sorting required and thus poses a challenge to systematic assessments [18]. To address this issue, many studies have employed bibliometrics, meta-analyses, or reviews to handle the large amount of literature. A large amount of literature using bibliometrics has been published in succession since a bibliometric analysis can effectively extract the knowledge status, information, and trends of a discipline [19]. Janko et al. [20] discussed whether the climate change debates are good for science, based on the bibliometrics. Huang et al. [21] analyzed 747 academic studies related to climate change and carbon sinks, based on bibliometrics, to provide the trends and references for the research potential in this field. Zhang et al. [22] used bibliometrics to identify the major interests and provide a fresh perspective for future research directions in the field of bioenergy under climate change, based on 3050 articles published since 1999. Leal et al. [23] analyzed the current state and future direction of migration and climate change, based on 333 documents, using bibliometrics. The meta-analysis approach has also been widely employed to summarize the results of the existing literature [24]. For example, Li et al. [25] reported a systematic meta-analysis of the impact of climate change on cotton yield. Hu et al. [26] performed a meta-analysis to reveal the variance in the tolerance to climate change across the marine trophic levels. Additionally, a series of reviews have assessed the impact of climate change [10,17,27]. All of those studies provided policy-related implications for better climate change adaptation.

### 1.3. Research Gap

Nowadays, most of the existing studies are confined to specific topics [28,29,30], and an overview of the entire research field on the impacts of climate change is lacking [31]. Moreover, the spatial heterogeneity among climate change studies in China has been overlooked [32]. However, the imbalance spatially of climate change impacts deserves much attention, since it is of vital importance for studying the climate change impacts in such a country across large latitudes and longitudes [33]. Therefore, methods to handle large amounts of scientific text, while exploring the spatial differences, are urgently required. Vo-Thanh, H. et al. [34] and Safaei-Farouji, M. et al. [35] employed machine learning techniques to explore the efficiency of carbon storage, which can help to mitigate climate change effectively. This exemplified to us that technological innovations and machine learning techniques are beneficial in the systematic assessment of the literature on climate change impacts.

### 1.4. Aim and Contribution

To bridge the current research gap, this paper firstly used the text-based classification method, combined with literature mapping, to evaluate the possibilities of climate change impacts on different regions. Specifically, the keywords including “climate change” and “impacts” were queried in the China National Knowledge Infrastructure (CNKI) database from 2000 to 2022. A total of 39,339 Chinese academic papers and 36,584 Chinese master’s and doctoral theses were included. Based on the abstracts of these studies, we extracted the geolocation of each article, based on a machine-learning pipeline, and counted the frequency. Furthermore, we mapped the literature geolocations into spatial grid data, with a resolution of 50 km × 50 km, to show the spatial heterogeneity. Based on this resolution, one grid cell generally covered a county, which is the minimum area unit used in most of the studies. Moreover, the text-based classification method was employed to extract the literature pertaining to temperature and precipitation changes separately to obtain the temperature and precipitation maps. The exposure of the gross domestic product (GDP) and the population to the impact of climate change was also demonstrated. This study systematically assesses the impact of climate change in China, on a pixel scale, to ensure a comprehensive analysis and provide policy support for climate change adaptation. Also, the main contributions of this paper are highlighted as follows:

(1) To appraise the big literature on climate change impacts more efficiently;
(2) To study the spatial heterogeneity of the climate change impacts;
(3) To analyze the potential impacts of climate change on the population and economy.

### 1.5. Organization of the Study

**Figure 1:**
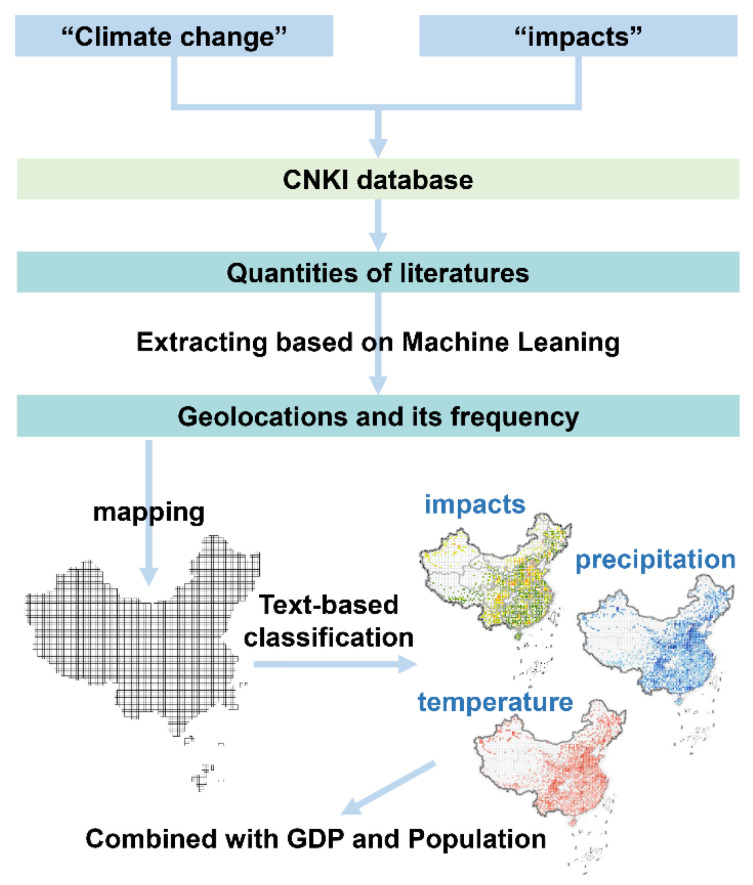
The overall framework of this study.

This paper is organized as follows: Section 2 illustrates the methods of mapping the studies of climate change, temperature change, and precipitation change impacts. In addition, the data sources of the GDP and population are also listed in this section. Section 3 discusses the results of the mapping of the literature and the impacts of climate change on the GDP and population in China. Section 4 summarizes the main conclusions and provides the policy implications. The overall framework of this study is presented in Figure 1.

## 2. Methods and Materials

### 2.1. Mapping Relevant Impact Studies

In this study, the terms “climate change” and “impacts” were used to query the CNKI database (accessed at https://www.cnki.net (accessed on 28 March 2022)) to systematically identify the literature, from 2000 to 2022, pertaining to the impact of climate change. All queried Chinese academic papers and Chinese master’s and doctoral theses were obtained (a total of 75,923 articles). Big data and machine learning techniques were used to process the massive literature and reduce the effort of manual sorting [36], combined with natural language processing (NLP) techniques, which are applied to assign text samples, such as abstracts, titles, and text, into one or more classes [18,37]. Then, a geoparser, pretrained [31] using neural networks, was used to extract the geographic information from the abstracts, text, and titles. Each geographic location was matched to a set of overlapping 50 km × 50 km grid cells. Thus, geographic maps of the impacts of climate change were obtained.

### 2.2. Classifying Studies on the Two Major Climate Variables

Changes in temperature and precipitation are the two main factors driving climate change [38,39]. The classification of studies based on these two variables is on a scale beyond that which can be done manually. The NLP technique extends the capabilities of text classification. In this study, a machine learning pipeline, following [18], was developed to classify and identify the studies on the impact of changes in temperature and precipitation on climate change. Specifically, the language model used in this study was a distilled version of the Bidirectional Encoder Representations from Transformers (DistilBERT) [40]. The DistilBERT model captures the context-dependent meaning of the text to represent the text [41]. Furthermore, the spatially explicit impacts of climate change by mapping the geolocation literature intuitively can show the hotspots of the current research, which is of great significance for mitigating and adapting the impacts of climate change based on local conditions.

### 2.3. Combining the Socioeconomic Data

The spatial GDP and population data were downloaded from the Resource and Environment Science and Data Center (https://www.resdc.cn/ (accessed on 28 March 2022)) with 1 km × 1 km resolution. The GDP layer grid data represents the specific spatial distribution of the GDP nationwide. The value of each grid cell is the total GDP within the grid cell (10,000 yuan/km^2^). The population dataset describes the population distribution in China, using units of 10,000 persons/km^2^. To be consistent with the impact of the climate change map, the GDP and population layers were resampled to the grid cell with 50 km × 50 km resolution in ArcGIS by using the method of nearest resampling. Note that the value of each grid cell of the resampled GDP and population layers represents the gross GDP and population within each pixel.

## 3. Results and Discussion

### 3.1. Maps of the Impacts of Climate Change

**Figure 2:**
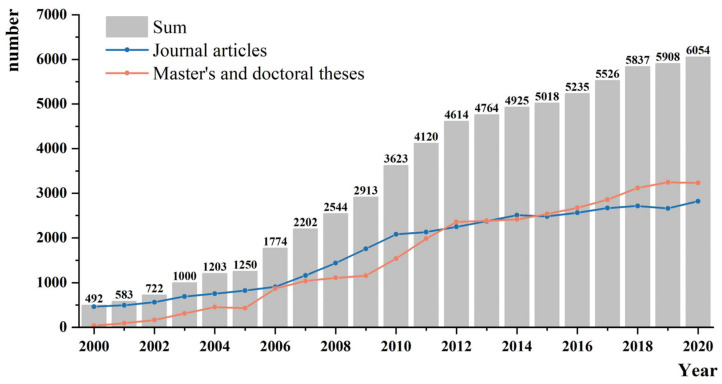
Growth in the number of articles on the impact of climate change over the past two decades.

Studies on the impacts of climate change have exploded with huge spatial heterogeneity in China during the last two decades (Figure 2). This increase in the studies indicates that increasing attention is being paid to research on the impacts of climate change. It may imply an intensified impact of climate change in China in recent years.

**Figure 3:**
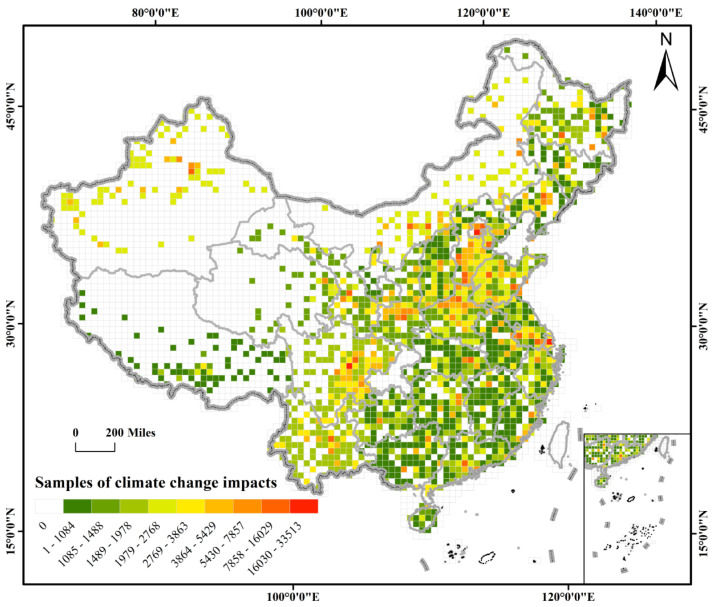
Geographic map of the impact of climate change studies.

Aiming to identify the climate change hotspots, we extracted the geolocations of more than 70,000 studies on the impacts of climate change and mapped them into grids with a resolution of 50 km × 50 km (Figure 3). Our results show that more than one-third (36.09%) of China’s land area has been studied for at least one aspect of climate change. However, the number of relevant studies in each province varied greatly. We divided the number of samples in each grid cell into nine levels. Specifically, a few scattered studies have been conducted in western and northern China, whereas sampling in eastern coastal China and southern China covered almost all the grid cells in these regions. Note that the white cells do not indicate the lack of an impact of climate change but, rather, areas where no publications were detected within those grids during the study period. However, in this study, the larger number of samples on the impact of climate change in one cell grid suggests a greater impact in that grid, at a regional scale.

**Figure 4:**
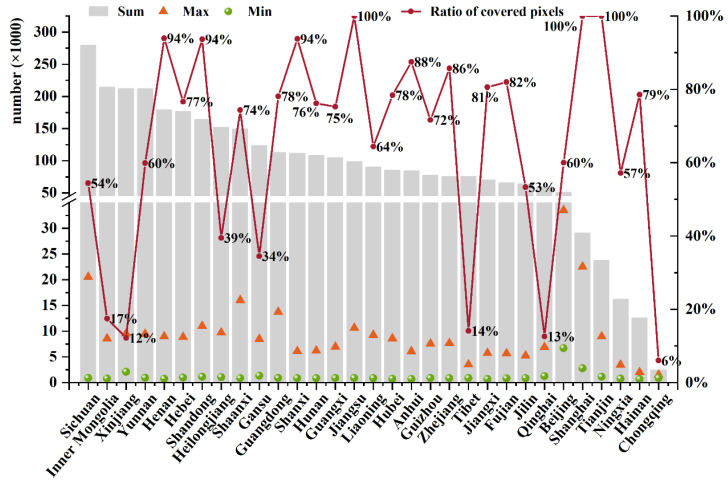
Summary of samples in each province and the ratio of covered pixels in the total area of each province.

The mapping revealed spatial differences at the provincial level. The developed areas and grain-producing areas in China have been studied more extensively. In particular, the Beijing–Tianjin–Hebei (BTH) urban agglomeration and Yangtze River Economic Zone have received considerable attention, comprising a strikingly high proportion of all the study cases in China. For example, 33,513 climate change impact study samples, the largest number among all the grids, were found in a grid in Beijing (Figure 4). In addition, Shanghai and Jiangsu Province, which are located in the Yangtze River Economic Zone, and Tianjin, located in the BTH urban agglomeration, may also be highly affected by climate change, because 100% of their area has been studied extensively. A total of 176,340 samples were found in Hebei, with 100% of its area covered. As the political, economic, and research center of China, it is reasonable that more literature and research are concentrated in these two regions. Additionally, another possible reason is that the BTH urban agglomeration is a major wheat-producing area, and the Yangtze River Economic Zone is the main rice-producing area in China. Agricultural production is highly sensitive to climate change; thus, evaluating the impact of climate change on agriculture is important because of the immense food demand in China. Similarly, other agricultural production areas have been well studied. For example, 93.65% and 93.94% of the grids in the grain-producing areas of Henan and Shandong, respectively, were classified as being extremely highly studied regarding climate change. The sample numbers in the two provinces shared 10.46% of all the samples in China, with a minimum sample number of grid cells in Shandong of more than 1000 (*n* = 1122). These studies suggest that almost all areas in these two provinces have a higher possibility of climate change risk. Similarly, the southwest agricultural production areas are other hotspot areas for climate change, as typical grain-oil-producing areas [42]. Sichuan Province may be the most affected by climate change, with the largest number of samples, (*n* = 279,500), accounting for 8.52% of all the samples in China, and 20,545 samples were found in one grid cell in this province. However, there was greater geographic variation in Sichuan than in other provinces. The differences in altitude explain the spatial heterogeneity in the climate change impact in this region.

Fragile ecological zones have also been investigated due to their vulnerability to climate change. Many studies were found in areas such as Yunnan, Xinjiang, and Inner Mongolia, with extremely large numbers of climate change samples (*n* = 211,760, 212,399, and 214,246, respectively). In particular, Xinjiang and Inner Mongolia are plateaus and major livestock production areas; thus, degenerating grassland and desertification are the major threats to these regions [43]. Yunnan has a high species richness, due to the complex terrain and dry hot valley climate [44]. Nevertheless, there were huge spatial differences, particularly between Xinjiang and Inner Mongolia. The grid cells possibly affected by climate change in Xinjiang were located mostly in the northwest, and only 12.20% of the grid cells showed the evidence. Furthermore, the minimum number of samples in a studied grid cell was as high as 2117 in Xinjiang. Similarly, 17.47% of Inner Mongolia has been studied. The grids with more samples in these regions were generally located in the urban areas, indicating that more work has been performed in areas with denser populations and more developed economies. However, we found no relevant studies in most other areas of these provinces, and the number of samples in the urban areas is markedly lower. Considering the wide-ranging impacts of climate change, it is unlikely that these white, or less-studied, grids are not affected by the impacts of climate change. Limited research sources may be available in those regions. The ecosystems in those regions are very fragile [45]; thus, more attention should be paid to untouched areas to better protect their vulnerable but rich ecosystem services.

### 3.2. Temperature and Precipitation as Representative Impact Maps

**Figure 5:**
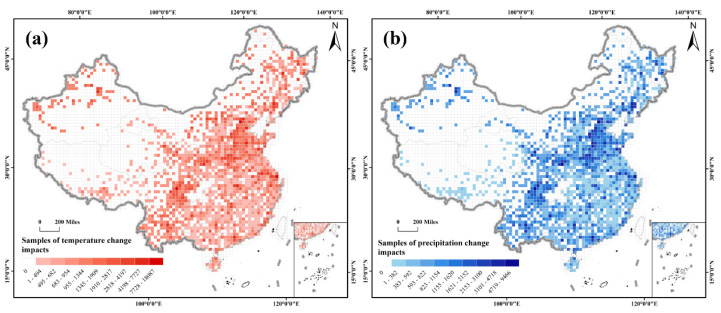
Maps of the impacts of changes in temperature and precipitation. (**a**) Map of the impact of changes in temperature. (**b**) Map of the impact of changes in precipitation.

Precipitation and temperature are the two main climatic factors [46]. In this study, we extracted the literature pertaining to precipitation and temperature separately, using NLP techniques, and mapped them into grid cells (Figure 5). The regions with more samples for temperature or precipitation changes were the research hotspot areas for the impact of climate change. The number of samples in each grid cell was divided into nine levels to represent the level of each pixel using Jenks’s natural breaks [47], a method that splits the data into statistically homogenous groups to minimize the intra-group variation and maximize the inter-group separation [48]. The details of these levels are shown in Figure 5. Levels 1–9 successively indicate the lower to higher levels of the impact of climate change variables that one grid cell may face (Figure 5).

**Figure 6:**
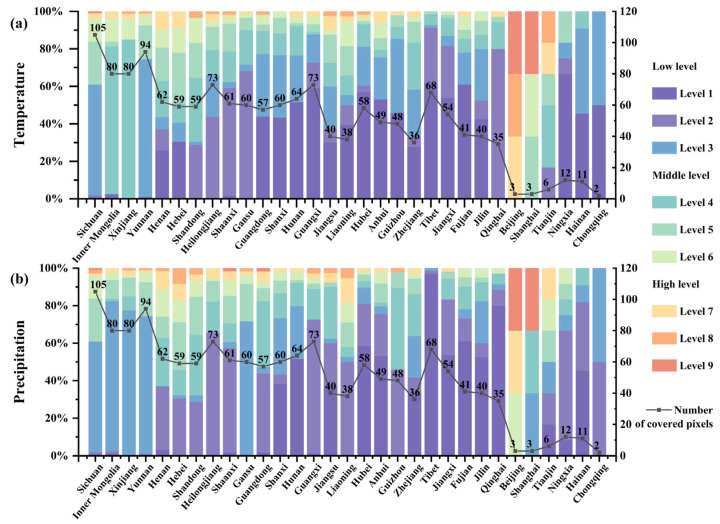
The percentages of samples for the impact of temperature and precipitation changes in 31 provinces. (**a**) The percentage of samples for the impact of temperature change in each province. (**b**) The percentage of samples for the impact of precipitation change in each province.

The challenges posed by the impact of temperature changes may be more difficult in China than those posed by the impact of precipitation changes in recent decades. There were 21.90% more samples concerning the impact of temperature change than the impact of precipitation change. The ratios of the grid cells at the different levels of temperature and precipitation change impacts in the cells of each province are shown in Figure 6. The developed regions, including Beijing, Tianjin, and Shanghai, face a higher risk of an impact from temperature change than precipitation change. The proportion of pixels with middle (levels 4–6) or high (levels 7–9) risk levels for temperature change in all the grid cells is comparatively high in the developed regions. This finding suggests that the urban heat island effect in developed regions has become a major threat due to rapid urbanization [49]. Similarly, Xinjiang and Inner Mongolia face greater challenges from the impact of temperature change than precipitation change. A strikingly high proportion of the grid cells is at the middle-risk level for the impact of temperature change (96.25% and 93.75% in Xinjiang and Inner Mongolia, respectively). Relatively larger proportions (77.50% and 82.50% in Xinjiang and Inner Mongolia, respectively) of the grid cells are at a low-risk level (levels 1–3) for the impact of precipitation change in the two provinces (Figure 6b). The two grain-producing areas, central and eastern China (Henan and Shandong) and northeastern China (Heilongjiang, Jilin, and Liaoning), face equivalent impacts from the changes in temperature and precipitation. The same trend in the proportion of pixels at different risk levels for the impact of temperature and precipitation changes is found in the other regions. A tailored package of measures concerning temperature change should be taken to relieve the corresponding impacts. Moreover, the impact of precipitation change cannot be ignored to prevent extreme precipitation events.

### 3.3. Potential Impact on the Population and Economy

The evidence-based assessments of the observed climate change impacts have been performed by the IPCC [50]; thus, we deduced that the regions with more research evidence have a larger possibility of being confirmed as having experienced larger climate change and facing a larger risk of the impacts of climate change. Furthermore, the quantity of research has concluded that the shift in temperature and precipitation patterns has caused huge economic damage [51,52]. Meanwhile, it has also posed threats to human lives and health, including mortality and morbidity [53,54]. It is impossible to numerically quantify the effects of the two changing climate variables in this study. Instead, this study evaluated the strong evidence demonstrating the size of the economy and population exposed to temperature and precipitation change, which can provide policy implications for climate change mitigation at a spatial scale.

**Figure 7:**
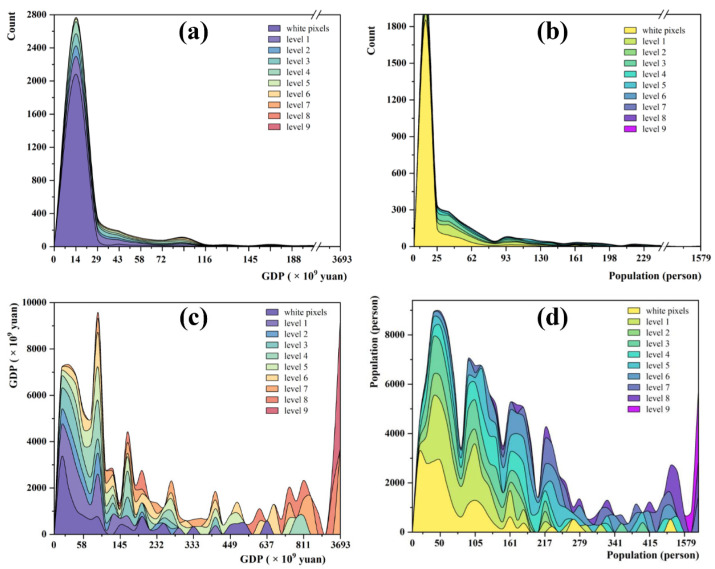
The distribution of GDP and the population in each grid cell at different levels of climate change impact and the corresponding sum of the GDP and population in each grid cell at different intervals. (**a**) The number of pixels at different GDP intervals. (**b**) The number of pixels at different population intervals. (**c**) The sum of GDP in each grid cell at different intervals. (**d**) The sum of the population in each grid cell at different intervals.

The grid cells at a higher risk of being affected by climate change were concentrated in the areas with a larger GDP and denser population in China. The distributions of the GDP and population in each grid cell are shown in Figure 7. The pixels with a low GDP and population were predominant (Figure 7a,b) at all levels of climate change impact. The sum of the GDP and population in each grid cell at different intervals (Figure 7c,d) suggests that a higher impact of climate change plays a more important role as the GDP and population increase in each grid cell. Although the number of grid cells with a large GDP and population is small, the sum of the GDP and population is not. This finding indicates that the center of the economy and the population usually face a higher risk from the impact of climate change.

**Figure 8:**
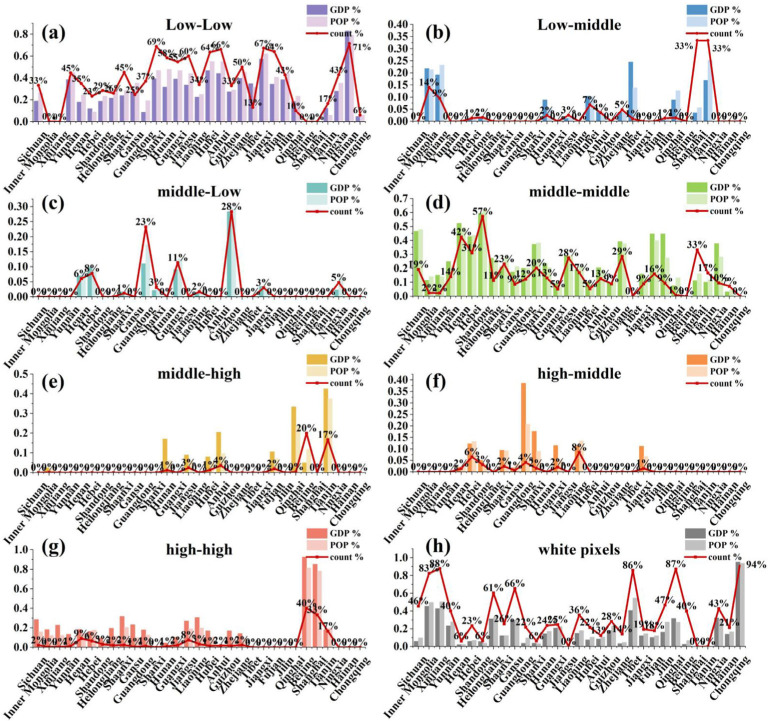
The proportions of the numbers of different types of grid cells in each province and the proportion of the GDP and population of those grid cells in each province.

The impacts of changes in temperature and precipitation on humans and the economies of provinces with large agricultural and developed metropolises have been well documented. To explore which climate variable has the greatest impact on one grid cell, we compared the two variables. The proportions of the grid cells at different levels of the samples on the impact of changes in temperature and precipitation, and the ratio of the GDP and population in each province, are shown in Figure 8. “Low-low” means the pixels at the lowest level of samples for the impact of temperature change and the lowest level of samples for the impact of precipitation change. The white pixels indicate that no samples were detected during the study period. Note that none of the grid cells were of the “low-high” or “high-low” types, and no grid cells were affected by a change in only temperature or only precipitation. This provides implications for assessing whether one province should receive more attention, due to its highly dense population and thriving economy.

The gap between the ratio of the number of pixels for the different types, and the proportion of GDP and the population in a province, can guide researchers to adjust their attention according to the socioeconomic status of a particular area. Shandong and Henan are the major populated provinces, and their GDPs rank highly in China. The grid cells of the “middle-middle” type in Shandong and Henan play a dominant role, accounting for 57.14% and 42.42% of the total number of pixels in Shandong and Henan, respectively (Figure 8d). Approximately half of the populations of Shandong (62.07%) and Henan (49.19%) are located in these grid cells. The GDP of these grid cells accounts for 59.34% and 52.35% of the total GDP in Shandong and Henan, respectively. This finding indicates that the impact of the changes in temperature and precipitation on human activities and economic losses in these two provinces has been realized. Beijing and Shanghai, as the economic centers of China, have been greatly affected by climate change. Approximately one-third of the pixels are of the “high-high” type (40.00% in Beijing and 33.33% in Shanghai), and this type of pixel contributes to the extremely high GDP ratio of 92.53% and 85.24% in Beijing and Shanghai (Figure 8g), respectively. This result suggests that it is reasonable to pay attention to those pixels and that taking measures in those areas can obtain multiplier effects for socioeconomic development. Additionally, the “middle-middle” type pixels in Sichuan, Fujian, and Jilin have high proportions of the GDP and population. The “low-low” type grid cells play the predominant role in most of the provinces (Figure 8a).

However, there are still untouched areas in climate change research in recent decades. The studies on the impacts of changes in temperature and precipitation in Chongqing are barren, but its population and GDP cannot be ignored. Relatively large sections of the population and GDP in the well-studied regions were represented by white pixels, indicating no climate change samples. For example, 82.53% and 87.70% of the grid cells have no temperature or precipitation samples but contribute 45.52% and 42.84% of the GDP in Inner Mongolia and Xinjiang, respectively. Although the proportions of the Sichuan population and GDP located in the white pixels are relatively low, their amounts are relatively large in China. This problem has not been addressed in studies on the impacts of climate change. The vulnerability to the impact of climate change in these regions, and the relationships with its population and economy, have imposed huge pressures on sustainable development, and it is of vital strategic significance to fully discuss the impacts of climate change.

## 4. Discussion and Policy Implications

The challenges posed by the impact of climate change in China are increasing. Fully utilizing the existing literature on the impacts of climate change will help provide a comprehensive view to adapt to future effects. Our results reveal a clear increase in the number of climate change impact studies over the past two decades. We show that more than one-third of the land area (36.09%) in China has been well studied, but the sampling was extremely imbalanced spatially. In this study, the economic, agricultural, and ecological centers attracted attention due to their important strategic roles. Extensive studies on the impacts of climate change have typically been performed in developed regions, such as the BTH urban agglomeration and Yangtze River Economic Zone, major agricultural production areas such as Shandong, Henan, and Sichuan, and ecologically fragile regions such as Yunnan, Xinjiang, and Inner Mongolia. Additionally, this study identified the dominant climate variables (temperature and precipitation change) in a region, which will help us better adapt to extreme climate events. The challenges posed by the impact of temperature changes may be grimmer in China than the precipitation changes in recent decades, particularly in the economic centers and ecologically sensitive zones. Nevertheless, there are still untouched research areas, particularly in Chongqing and non-urban areas of the ecologically sensitive zones.

The current research status of the impacts of climate change is closely related to the regional variations in the population, economic development, and research resource distribution in China. The research institutions are concentrated in regions with denser populations and thriving economies. Focusing on these regions has crucial implications for economic development, crop-planting structural adjustment, grain yield and quality improvement, and ecological service conservation. While repetitive studies in one region are not advisable, China should offer more funding for studies on the impacts of climate change in ecologically sensitive and rural regions. Paying attention to those areas with rare research sources would provide a more comprehensive perspective to mitigate and adapt to the impacts of climate change.

This paper has several limitations, as follows: Firstly, more extensive literature from multiple bibliographic databases is required to provide further evidence on the impact of climate change in China. Secondly, more climate variables can be considered to cast light on comprehensive attribution mapping to provide an integrated view of climate change adaptation. In future research, machine learning techniques can support the researchers in depicting a more convincing attribution mapping of the big literature. However, this study provides a basic and pioneering attempt to evaluate the possibilities of climate change impacts on different regions.

## 5. Conclusions

Extensive studies on the impacts of climate change are concentrated in developed regions, major agricultural production areas, and ecologically fragile regions. This indicated that these areas may face a more challenging risk of climate change impacts. Thus, it is of great importance to take measures to release the negative impacts of climate change for economic development and human well-being. Meanwhile, this showed the spatial imbalance of current research concerning climate change impacts. Therefore, paying attention to the ecologically sensitive and rural regions is advisable to provide a comprehensive perspective to mitigate and adapt to the impacts of climate change.

## Data Availability

Not applicable.
